# Constructive Suggestions toward the Control of Syphilis, Gonorrhea, Pneumonia, Malaria, Typhoid, and Typhus Fever

**Published:** 1917-10

**Authors:** Ira S. Wile

**Affiliations:** New York; Associate Editor of “American Medicine.”


### CONSTRUCTIVE SUGGESTIONS TOWARD THE CONTROL OF SYPHILIS, GONORRHEA, PNUEMONIA, MALARIA, TYPHOID, AND TYPHUS FEVER.

**By Ira S. Wile, M.D., New York.**
Associate Editor of "American Medicine."

All programs looking toward the control of infectious diseases should be based upon the theory that control for prevention is superior to control for cure. While the development of hospital and dispensary methods plays a part in the elimination of infectious diseases, its importance varies largely according to the prevalence of specific diseases and the efficiency and social reactions of the medical institutions. From the standpoint of preventive control, the curative phases of control are of service only in so far as they effectively serve to eliminate known foci of infection. Were it possible to hospitalize immediately all patients during the incubation period and thus prevent the spread of disease during its incipiency, hospitals might afford a more practical solution of the control of infectious diseases. This possibility, however, is obviously limited, and in consequence hospitals and dispensaries now play a subordinate part in the control of syphilis, gonorrhea, pneumonia, malaria, and typhoid and typhus fevers.

The general ineffectiveness of hospitals and dispensaries in attacking the above mentioned diseases indicates that thus far they have played a comparatively small part in the reduction of their morbidity rate, though in such conditions as gonorrhea and syphilis their use would be of paramount advantage. Preventive control is necessary from the medical, economic, and social standpoint. Curative agencies must become instruments for prophylactic service.

In the discussion of prevention for control one immediately seeks the facts common to the group of diseases under analysis. It is patent that the two main elements in a program for prevention must involve personal and social plans. The hygiene and morality of individuals and their homes most assuredly require definiteness and intimate contact and more intensified programs than are involved in the general social measures requisite for municipal sanitation, public education, and social reformation. The methods to be utilized in the control of these diseases, however, may be general in character in so far as they involve the personal and social hygiene which underly all the infectious diseases. While malaria and typhus fevers are actually transmitted by insects, the mosquito and the body louse, and gonorrhea and syphilis are spread through direct personal contact, typhoid and pneumonia similarly present numerous instances in their etiology indicative of the importance of the personal and social factors for their control.

These diseases are responsible for a varied mortality which is inconsequential in the case of gonorrhea but is exceedingly serious in pneumonia. Far more important, however, is the morbidity from those various diseases with the resultant economic invalidism, social degeneration, and racial impoverishment. The direct mortality of gonorrhea and syphilis is excedingly small, but its indirect results are of paramount importance. Gonorrhea with its hideous trail of blindness sterility, and surgical mutilations presents a public health problem which is well understood by workers in charities and corrections as well as physicians. Syphilis with its dysgenic effects, including abortions, miscarriages, infant mortality, mental defects, and insanities, yields results which are all but concealed in the consideration of the statistics of its mortality.

In the order of relative importance, according to mortality, one would speak of pneumonia, typhoid fever, malaria, typhus fever, syphilis, and gonorrhea. From the public health standpoint, and indeed from the standpoint of social medicine, syphilis and gonorrhea, as far as New York city is concerned, should be placed practically at the head of the list.

During 1916 New York city was shocked at the occurrence of 9, 023 cases of poliomyeletis. There were only 208 cases of malaria and 215 of typhoid fever. Despite the continued campaign against tuberculosis there were 19,297 cases reported during the year. But who realized that there were 20,128 cases of syphilis and 6,220 cases of gonorrhear notified to the health authorities? There was no epidemic; there was no publicity; no excitement; no enthusiastic society seeking to secure their elimination. In corroboration of the statement that this occurrence of gonorrhead and syphilis is not unusual, let me call attention to the fact that during thirteen weeks of 1917 ending April 21st there were 1,535 cases of gonorrhea and 5,642 cases of syphilis. How rare typhoid fever appears with its 234 cases and typhus with its three cases during the same period of time! Even pulmonary tuberculosis with its 736 cases is completely overshadowed.

The first step in modern methods in controlling infectious diseases requires an arousing of the public mind to the importance of the problem to be attacked. There can be little enthusiasm aroused in a fight to combat malaria whose mortality rate has been decreased 86.5 per cent. during the ten years, 1900—1904 to 1910—1914. The discomfort arising from the pest of mosquitoes might start a health department in a war against the varied biting mosquitoes which contribute to personal discomfort though they are not responsible for malaria. The control of venereal diseases demands as great publicity and popular education as was employed by the health department in its recent attack upon pneumonia, influenza, and other respiratory diseases. Suggestions for preventive control must necessarily be brief and therefore must also be incomplete.

In fighting against malaria, in addition to the educational propaganda, there is involved the destruction of mosquitoes through drainage; the protection of the well from the bites of Anopheles through adequate home screening; the elimination of breeding places throughout the community. Add to this, the protection of those infected with malaria from mosquitoes, the establishment of diagnostic facilities for revealing the presence of the plasmodia in the blood, the availability of quinine for those actually ill, and the control of those who have suffered from the diseases with a view to preventing the breaking out of latent infection. In this plan the burden of responsibility rests upon the health department, aided by the intelligent cooperation of the medical profession. The elimination of Anopheles would reduce the personal responsibility for the continuance of the diseases almost to the vanishing point.

Since 1896 when Brill described the mild form of typhus fever so frequently called by his name, typhus has been endemic in New York city. The bacillus causing it has been discovered by Plotz, and our knowledge regarding the method of its spread and control has been enriched greatly through the experience during the past bloody months on foreign fields. Hirsch has well said, ''The history of typhus is the history of human wretchedness.'' The program for control involves the exceedingly difficult problem of the elimination of the body louse, Pediculus vestimenti, and the head louse, Pediculus capitis. These body pests are responsible for much discomfort and disease, and present a herculean task. Thriving in the midst of ignorance and poverty their habitat is readily accessible but none the less elusive. A plan for their control involves the entire problem of home sanitation, higher wages, and education, which may be wisely supplemented by the establishment of municipal laundries and disinfection stations for the proper care of infected clothing. The medical inspection of school children can be utilized, as it is in part, for indicating those homes requiring the most careful attention.

Concerning pneumonia few practical suggestions can be made which do not involve the socialization of communities. We are more familiar with the types of pneumonia and the various bacteria involved than we are with the fundamental facts underlying pneumonic infection. It has been recognized that the discharge from the nose and mouth are the main instruments in the dissemination of the disease. Correct breathing habits are not of maximum service, unless the vitality of the community is enhanced, and healthful conditions are established in the home and public places. The police power of the health department properly has been called into action to relieve the overcrowding of our transportation facilities. Overcrowding, however, must be prevented in churches, schools, theatres, and other private and public places where masses of the citizenry congregate. Streets must be clean and preferably oiled, while ashes require careful handling. Rational ventilation must become a household practice. Every case is a focus of distribution, and disinfection of the infective discharges becomes imperative. Isolation of the patient merits trial when practicable. On the other hand malnutrition calls for attention, and the wider installation of school lunches would offset the dire effects of underfeeding. The increase of social legislation tending to restrict the devitalizing influence of modern industries is no less important than is an increase in the living wage to make possible the maintenance of a decent standard of living compatible with vigorous resistance to disease.

So far as typhoid fever is concerned, the general experience in the United States army and elsewhere is thoroughly indicative of the fact that vaccination against typhoid fever is as important a health measure as vaccination against smallpox. The free distribution of antityphoid vaccine is to be urged, and the general public should be educated as to its importance, particularly that portion of the public financially able to take a vacation outside of the city limits. Hospitals and private physicians should report the typhoid carriers to permit the extension of control in connection with industrial hygiene. Such registration affords a point of departure in the protection of the community against typhoid carriers engaged in cooking, serving, handling, or producing foods. Pasteurization of milk in the bottles instead of by the holding process would further protect the community from the milk borne epidemics. The general public should be informed when the water supply is polluted and warned to boil all drinking water. It is conceded that food inspection and control must be advanced through state legislation and local ordinances to insure their cleanliness and safety. It is of the utmost moment that the watershed yielding the potable water supply be protected, and the muncipality should call to its support every social and medical agency in urging upon the legislature the necessity of modern safeguards against the pollution of the drinking supply of the community.

Gonnorrhea and syphilis may be considered as a single item inasmuch as the underlying causes are identical for the two diseases with the exception of their bacteriology. These diseases are accidental social infections arising from direct contact. A negligible proportion of them originate innocently. In the program for attacking these plagues there come to the fore many social problems, especially those related to prostitution. Concerning the question of segregation, legislation, and medical supervision little need be said, save that these methods have not proved effective in decreasing venereal disease and are distinctly opposed to the moral interests and ethical development of the community. Such methods as the use of the Injunction and Abatement Act, the registration and public advertisement of properties utilized for immoral purposes, the enforcement of existing laws and regulations tend to decrease this medium of contagion, but certainly cannot eliminate prostitution. Education, rationl recreation, higher wages, early marriage, better homes and privacy, the restriction of dance halls, the control of the sale of alcohol, raising the age at employment, and the improvement of the standards in industry are of the utmost importance in developing an environment which tends to short circuit sexual desire into normal and healthful channels. A campaign of education through the boy scout organizations, the camp fire girls, church societies, mothers' clubs of the community should be accelerated through properly chosen lecturers of the department of health cooperating with workers in social hygiene. A systematic protective education is essential to combat the traditions of the street and the unhallowed misinformation which is largely responsible for the widespread abuse of the sexual function which underlies venereal diseases.

In the face of a mobilization of soldiers it is important to stress the safeguards which must be placed about adult males unfettered from the restraining influences of home environment. The development of a bureau of recreation will probably be of the utmost advantage in offsetting the temptations of camp life. Despite all endeavors, including the banishment of prostitutes beyond a three mile limit of camps, there will undoubtedly be a large number of infections which will work to the physical degeneration of the men and later will visit afflictions upon the women to whom they may return as, or to become, husbands. From the standpoint of military efficiency the actual prevention of gonorrhea and syphilis is of greater consequence than the ethical aspects of the subject. Along with the constructive program of preventing venereal diseases in the army, consideration must be given to the utilization of the packet designed to prevent infection after exposure. The grimness of war and the necessity of maintaining military efficiency, although acceptance of this doctrine may cause a shock to those whose minds are dwelling upon the moral phases of venereal diseases without giving adequate consideration to the practical military health problems involved.

While the ethical and moral sides of the venereal problem are the main issues to be met from the standpoint of public health, an earnest endeavor must be made to counteract the existing epidemic of syphilis and gonorrhea. Large as are the figures of reported cases in this city, they underestimate the total number of cases and are particularly lacking in accuracy for gonorrhea. A program for the prophylactic control of these diseases must be constructed on the basis of the recognition of syphilis and gonorrhea as diseases of more serious consequence to the community than practically any other disease with the possible exception of tuberculosis. From the health viewpoint the mode of attack must not involve the stigmatization of the sufferers as moral outcasts or degenerates. Prevention necessarily involves the cure of the victim, who otherwise is a constant menace to the community as a focus of infection. Our hospitals and dispensaries treating these diseases have failed to justify themselves by their results. The percentage of their established cures is disgracefully small, being in the large majority of institutions under fifteen per cent. This means a low grade of efficiency which would scarcely be tolerated in any other field of human endeavor. To counteract these negative tendencies, dispensaries should be obliged to have a standardized equipment suitable to meet the needs of treatment. Hospital facilities should be available for incipient cases, without the necessity of segregation in special hospitals and without penalizing those infected because of the nature of the method of infection. From the standpoint of communal usefulness, hospitals can perform no greater service than by curing luetics and gonorrheics during the stage of greatest curability. It is reasonable to demand such service from all institutions receiving public money for caring for public charges. The community should be able to dictate the types of cases requiring hospitalization. If this can be done during poliomyelitis, epidemic, it certainly is within its jurisdiction for syphilis endemic.

The compulsory notification of venereal diseases is imperative for any plan of systematic follow up work designed to facilitate their control. Following upon the registration of patients there should be established diagnostic laboratories, together with development of a system of evening pay clinics. The physician in attendance at such pay clinics should be adequately remunerated out of the fees collected. At every genitourinary clinic there should be a follow up social worker to secure the regular attendance of the clientele of the clinic. Under existing conditions the municipality should supply, without cost, the amount of salvarsan or any remedy requisite to insure the public against the continued danger from infected individuals. With the development of the diagnostic and therapeutic clinics full benefit cannot accrue to the community unless indigents and those unable to pay for expensive and intensive medication actually receive it, and this can only be accomplished at the public expense.

A final step, or possibly the first step, to be taken is the utilization of every medium for publicity in order to arouse the public mind to the importance of venereal diiseases. Posters, placards, bulletins, lectures, paid advertisements if necassary, are splendid investments for public health and will break down the accursed policy of silence which has permitted these diseases to develop and spread without a satisfactory public counterattack. If the charlatan and quack can flourish on the basis of their well selected publicity there is no reason why more constructive forces of the community should not avail themselves of similar measures to combat these diseases. Printers' ink is cheaper than salvarsan. The need for legislation to eradicate quickery and fraudulent advertisements is too obvious to require discussion.—New York Medical Journal.

